# THE UNCANNY VALLEY REVISITED: AGE-RELATED DIFFERENCE AND THE EFFECT OF FUNCTION TYPE

**DOI:** 10.1093/geroni/igz038.1202

**Published:** 2019-11-08

**Authors:** Yun-Chen Tu, Sung-En Chien, Yueh-Yi Lai, Jen-Chi Liu, Su-Ling Yeh

**Affiliations:** 1 Department of Psychology, National Taiwan University, Taipei City, Taiwan; 2 Intelligent Vision System Division, Electronics and Optoelectronics System Research Laboratories, Industrial Technology Research Institute, Hsin-Chu County, Taiwan

## Abstract

Due to declined birthrate and the increased aging population, solving the problem of labor shortage has become important. Introducing robotic labors could effectively help older adults’ daily lives. However, older adults’ acceptance of robots was lower than younger adults. Robot’s appearance might be one of the reasons. The Uncanny Valley (UV) refers to the phenomenon that people rate more positively as robots become more humanlike, but only up to a certain point; as it approaches near-perfect similarity of human appearance, likeability drops and forms an uncanny valley. Nonetheless, previous results supporting the UV were mainly from younger adults. We examined whether the UV is also applicable for older and middle-aged adults. We also examined whether the acceptance of function (companion vs. service) would change based on robot appearance, and whether robot-induced traits have any relation with the acceptance of robot function. We asked younger (N= 80, age 18-39), middle-aged (N= 87, age 40-59), and older (N= 88, age 60-87) adults to view each picture of 84 robots and evaluate their impression of each robot and intention of use regarding robot function. Contrary to the UV found for younger and middle-aged adults, older adults did not show UV–they preferred humanlike over non-humanlike robots, regardless of the robot function. Scores on each trait–except for authoritativeness–showed positive correlations with the acceptance of functions. These findings imply that the design of assistive robots should take UV into consideration by customizing robots’ appearances and functions to different age groups.

